# Molecular Epidemiology of Turkey Coronaviruses in Poland

**DOI:** 10.3390/v14051023

**Published:** 2022-05-11

**Authors:** Katarzyna Domańska-Blicharz, Anna Lisowska, Justyna Opolska, Anna Pikuła, Joanna Sajewicz-Krukowska

**Affiliations:** Department of Poultry Diseases, National Veterinary Research Institute, Al. Partyzantów 57, 24-100 Pulawy, Poland; anna.lisowska@piwet.pulawy.pl (A.L.); justyna.opolska@piwet.pulawy.pl (J.O.); anna.pikula@piwet.pulawy.pl (A.P.); joanna.sajewicz@piwet.pulawy.pl (J.S.-K.)

**Keywords:** turkey, coronavirus, Poland

## Abstract

The only knowledge of the molecular structure of European turkey coronaviruses (TCoVs) comes from France. These viruses have a quite distinct S gene from North American isolates. The aim of the study was to estimate the prevalence of TCoV strains in a Polish turkey farm during a twelve-year period, between 2008 and 2019, and to characterize their full-length S gene. Out of the 648 flocks tested, 65 (10.0%, 95% CI: 7.9–12.6) were positive for TCoV and 16 of them were molecularly characterized. Phylogenetic analysis showed that these strains belonged to two clusters, one formed by the early isolates identified at the beginning of the TCoV monitoring (from 2009 to 2010), and the other, which was formed by more recent strains from 2014 to 2019. Our analysis of the changes observed in the deduced amino acids of the S1 protein suggests the existence of three variable regions. Moreover, although the selection pressure analysis showed that the TCoV strains were evolving under negative selection, some sites of the S1 subunit were positively selected, and most of them were located within the proposed variable regions. Our sequence analysis also showed one TCoV strain had recombined with another one in the S1 gene. The presented investigation on the molecular feature of the S gene of TCoVs circulating in the turkey population in Poland contributes interesting data to the current state of knowledge.

## 1. Introduction

Turkey coronavirus (TCoV) is the etiological agent of an acute, highly contagious disease of turkeys known as bluecomb disease, mud fever, transmissible enteritis, or more recently, turkey coronavirus [1]. The disease is characterized by enteritis, anorexia, diarrhea, growth depression, retarded development, impaired feed conversion, and sometimes increased mortality. Due to these adverse effects, the virus is responsible for significant economic losses in the turkey industry. The disease, caused by TcoV, was first described in the 1950s, although the etiological agent was not identified until 1970. TCoV is reported worldwide, in different regions of the USA and Canada and also in South American and European countries, and in Australia [2,3,4,5,6]. It belongs to the *Igacovirus* subgenus within the *Gammacoronavirus* genus (*Nidovirales* order, *Cornidovirinae* suborder, *Coronaviridae* family, *Orthocoronavirinae* subfamily) together with similar viruses isolated from other domesticated Galliformes, including the most known infectious bronchitis virus (IBV). The official taxonomy divides these viruses into two viral species: avian coronavirus (AvCoV) and avian coronavirus 9203 (AvCoV9203), which together cover all IBV genotypes and turkey coronaviruses [7]. They belong to the same species due to similar phylogenetic relationships and genomic structures. Both viruses’ genomes are approximately 27 kb long single-stranded, positive-sense RNA consisting of 15 nonstructural proteins (nsp2–16) encoded by open reading frame (ORF) 1a/b at the 5′ end, which is associated with RNA replication and transcription, followed by the structural proteins spike (S), envelope (E), membrane (M), and nucleocapsid (N) encoded by other ORFs at the 3′ end. The S glycoprotein is post-translationally cleaved into S1 and S2 subunits during viral maturation. The S2 subunit anchors the spike into the virus membrane, whereas S1 forms the extracellular part of the spike and plays a major role in tissue tropism [8]. The 3′ end of the genome also encodes a few low molecular accessory proteins (3a, 3b, 4b, 4c, 5a, 5b and 6b), whose number and nature vary depending on the species and even the AvCoVs strain [9,10,11,12]. Turkey coronavirus and IBV also have a close genetic relationship given that the nucleotide similarity of whole TCoV and IBV genomes is about 86%. It is the S glycoprotein that most distinguishes the two viruses, as it shares only a 36% identity [9,13]. The different S genes affected the tropism of the virus, as IBV has an affinity to the respiratory/renal system and TCoV to the digestive system [14,15]. Most probably, the S gene of TCoV originates from an unknown coronaviral donor acquired during a recombination event [9,16]. It cannot be ruled out, however, that the S gene donor was not the avian coronavirus, as turkeys appear to be susceptible to infection as well as mammalian coronaviruses [17,18]. Bovine betacoronavirus could infect turkey poults, leading to fecal virus shedding, diarrhea, seroconversion and transmission to contact birds [17]. Similarly, in porcine deltacoronavirus-inoculated turkey poults, diarrhea, virus in cloacal and tracheal swabs as well as specific antibody responses, and antigen-positive cells in intestines were observed [18]. Moreover, deep molecular studies suggest that European and North American TCoV strains have different evolutionary pathways in both continents [9,16].

Our previous investigation revealed the prevalence of TCoV in the turkey population in Poland at a level of 7.3–9.7% [19]. In 2016, the presence of an atypical TCoV strain was also detected. Molecular studies of a complete genome sequence of TCoV isolated from the duodenum of turkeys suffering from acute enteritis revealed its unique characteristics: the genomic backbones of IBV GI-19 lineage and the S gene related to the North American TCoVs and French guinea fowl coronaviruses [20]. The objective of the present study was to estimate the relationship between the TCoV strains detected in a Polish turkey farm during a twelve-year period, between 2009 and 2019, by the full-length S gene.

## 2. Materials and Methods

### 2.1. Ethics Statements

Sampling was done with the consent of the owners or other responsible persons. The turkeys on the farms were under the supervision of veterinarians who took various samples as part of their routine work (e.g., as samples to test for the presence of any infection) and some of these samples were used in this study. For this reason, the collection of samples did not require approval from the Ethics Committee.

### 2.2. Sample Collection and Preparation

From 2008 to 2011, fecal swabs were collected as part of regular monitoring in which samples were collected from turkey flocks, regardless of their health status. The samples were tested in the later period, from 2012 inclusive, and were sent to the laboratory for diagnostic purposes (detection of enteric viruses, i.e., rotaviruses, parvoviruses, astroviruses and coronaviruses) from flocks showing symptoms of enteritis. During a twelve-year period (2008–2019), clinical samples from a total of 648 turkey flocks were collected from different regions of the country, mostly from the north-eastern and western parts of Poland, where about 70% of the Polish turkey industry is located. The samples originated from turkeys between 1 to 155 days of age. All samples were stored at −20 °C until processing. After slow thawing, samples from individual flocks were suspended *w*/*v* and ground in phosphate-buffered saline. The suspensions were centrifuged at 3000× *g* for 15 min, and 200 µL of obtained supernatants were used for nucleic acid isolation.

### 2.3. RNA Isolation

Total RNA was extracted from all field samples with the RNeasy Mini Kit (Qiagen, Hilden, Germany) following the manufacturer’s procedure.

### 2.4. Coronavirus Detection

The presence of turkey coronavirus was determined by two methods. At the very beginning (2008), amplification of the conserved region of the 3′ untranslated region (UTR) of the IBV genome was carried out by reverse transcription-polymerase chain reaction (RT-PCR) according to Cavanagh et al. [21]. Later, the real-time RT-PCR (rRT-PCR), aimed at 5′ UTR, was used [22]. Both methods were conducted on the one-step model using the One-Step or QuantiTect RT-PCR kits (Qiagen, Germany) according to the manufacturer’s instructions.

### 2.5. TCoV S Gene Amplification and Sequencing

For samples with high virus loads (Ct values between 16 and 25), we also applied various combinations of primers that specifically amplify the S gene previously described, or that were kindly provided by Dr. Nicolas Eterradossi (Anses, Ploufragan, France), as well as additional primers that were specifically constructed for some strains [3,11]. The reactions were run according to the recommended protocol for the kit (One-Step RT-PCR Kit, Qiagen, Germany) with different annealing temperatures depending on the melting temperature of the primer pair used. PCR products were sequenced in the commercial service Genomed Sp. z o.o. (Warsaw, Poland) in both directions, using Sanger sequencing technology. The complete S gene sequences of the Polish TCoV strains were manually assembled from 7–8 overlapping gene fragments by using the Geneious software (Biomatters Ltd., Auckland, New Zealand).

### 2.6. Sequences Analysis

To investigate the phylogenetic relationship of the obtained viruses, different CoV sequences were downloaded from GenBank, including the North American and French TCoVs, GfCoV and atypical IBV from China. All phylogenetic analyses, including the percentage of nucleotide and amino acid sequence similarities, were performed with the Geneious software (Biomatters Ltd., New Zealand). Phylogenetic analysis was performed using the maximum likelihood method and best-fitting substitutions models with MEGA v11 software. Bootstrap analyses of the resultant trees were performed using 1000 replicates [23]. 

The nucleotide sequence of the S gene was also deduced into amino acids and analyzed for the presence and pattern of a peptide cleavage site separating the amino-terminus of the S1 subunit from the carboxyl terminus of the S2 subunit, as well as a second possible peptide cleavage site in the S2 subunit (https://services.healthtech.dtu.dk/service.php?ProP-1.0 accessed on 10 April 2022).

To detect any recombination events in the Polish TCoVs, the S sequences were analyzed using the RDP, Geneconv, Maxchi, BootScan, 3Seq and Chimaera methods available in the RDP package v.4 [24]. Only the recombination events that were identified by at least three different methods and with a *p*-value below 1.0 × 10^−10^, were taken into account.

To check if individual codon sites in the whole S gene of the detected TCoV strains are subjected to positive or purifying selection pressure, an analysis was carried out using various bioinformatics tools from the Hy-Phy package (www.datamonkey.org accessed on 11 April 2022). The ratio of non-synonymous (dN) to synonymous (dS) nucleotide substitutions per site (dN/dS) and the selection pressures using the methods for individual codons were estimated (Fixed-Effects Likelihood—FEL; Fast Unconstrained Bayesian Approximation—FUBAR; Single-Likelihood Ancestor Counting—SLAC; Mixed Effects Model of Evolution—MEME). Positively selected sites were only confirmed by at least two different methods.

## 3. Results

### 3.1. Turkey Coronavirus Prevalence

During the period of 12 years, out of the 648 flocks tested, 65 (10.0%, 95% CI: 7.9–12.6) were positive for TCoV (Table 1). The highest number of samples was collected in 2008 and 2014 (80 and 77 flocks, respectively) and the lowest in 2010–2011 (32 and 39 flocks, respectively). The prevalence of TCoV varied with the year of the study and was highest in 2010 and 2019, when 9–10 positive flocks were detected, and lowest in 2011 and 2013 when none or only one infected flock was identified. In the remaining years, on average, 5–7 positive flocks were detected per year.

### 3.2. Full S Gene Analysis

The sequence of the whole S gene was obtained for 16 detected field TCoV strains, which were submitted to the GenBank database, and the accession numbers assigned are presented in Table 2.

**Figure 1:**
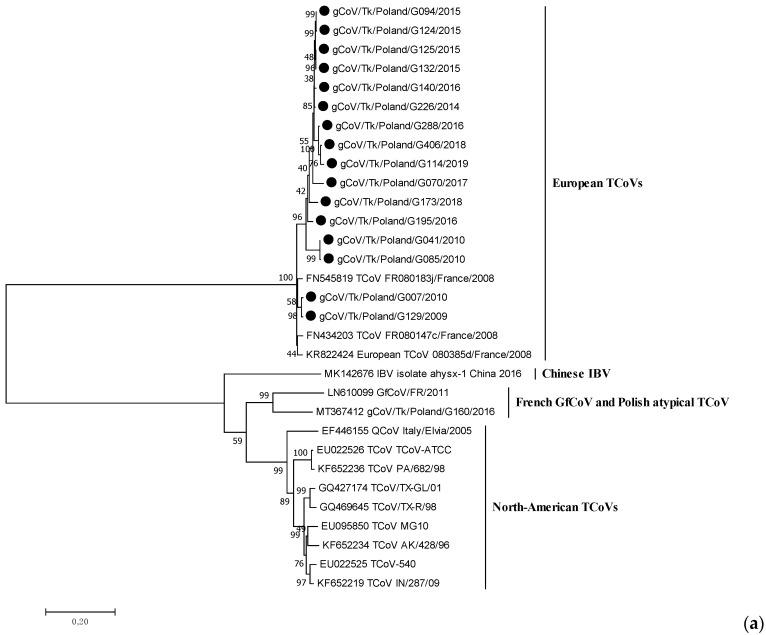

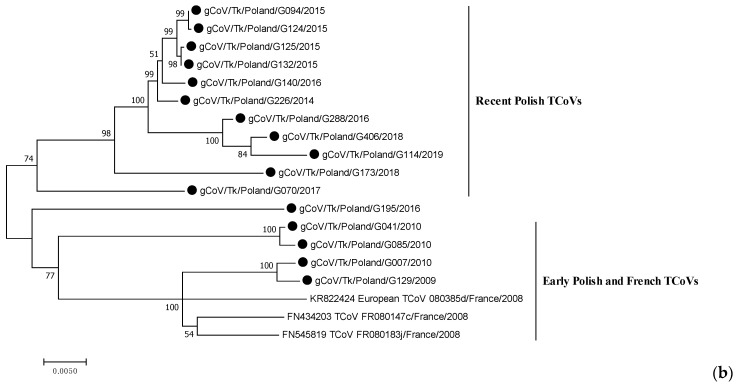
Phylogenetic relationships based on ML tree constructed from the full S gene of Polish TCoV strains with other similar avian coronaviruses (**a**) and only European TCoVs (**b**). Polish TCoV strains are marked with a black dot.

The length of the obtained S genes of the Polish TCoV strains ranged from 3424 (incomplete sequence of G070/2017 and G173/2018) to 3600 nucleotides. All isolates exhibited a similar structure to this gene. A phylogeny based on the complete S gene sequences showed that the Polish strains from 2009 to 2019 were on the same branch as the coronaviruses isolated from turkeys in France, but differed from North American TCoV strains and coronaviruses detected in guinea fowl in France, with the only one recently identified in turkeys in Poland (Figure 1a). Polish strains were clustered into at least two branches supported by bootstrap values >70% that corresponded clearly to the isolation period (Figure 1b). Group 1 (bootstrap value of 74%) comprised ten strains from the years 2014–2019 identified in Warminsko-Mazurskie province (six TCoVs), Mazowieckie (three strains) and Lubuskie (one strain). Group 2 (bootstrap value of 77%) contained four early TCoVs detected between 2009 and 2010: two strains were identified in Warminsko-Mazurskie in 2010 and two TCoVs were collected in Wielkopolskie in 2009–2010. The viruses from this group were mostly related to the French TCoV strains from 2008. The strain G195/2016, with an undetermined place of origin, did not belong to any of these two groups and formed an independent branch of the phylogenetic tree.

Sequence analysis of the full S gene revealed that Polish TCoV strains shared a nucleotide identity of 94.3–100%. Nucleotide homology between strains of Group 1 was 96.5–100% and between them and the European reference 080385d/2008 strain, it was 94.7–96.6%. In turn, the similarity of TCoVs from Group 2 was 95.2–99.5% and showed a 95.8–98.2% identity when compared with the European reference strain 080385d/2008 (Table 3).

The analysis showed that the strains from the same year and region were the most similar (G132/2015 and G125/2015 from the Warminsko-Mazurskie voivodeship), while strains from the most distant year of collection, 2009 and 2019 (G129/2009 and G114/2019), were the most different. All Polish TCoVs shared a 94.7–97.2% nt identity with the European reference TCoV strain, FR080385d. The next gammacoronaviruses with high similarity to the Polish TCoVs were the North American TCoVs, with a nucleotide sequence identity of 62.3–64.1%. The nucleotide identities with GfCoV/FR/2011 and gCoV/Tk/Poland/G160/2016 ranged from 61.9 to 63.9% and 61.4 to 62.9%, respectively.

**Figure 2:**
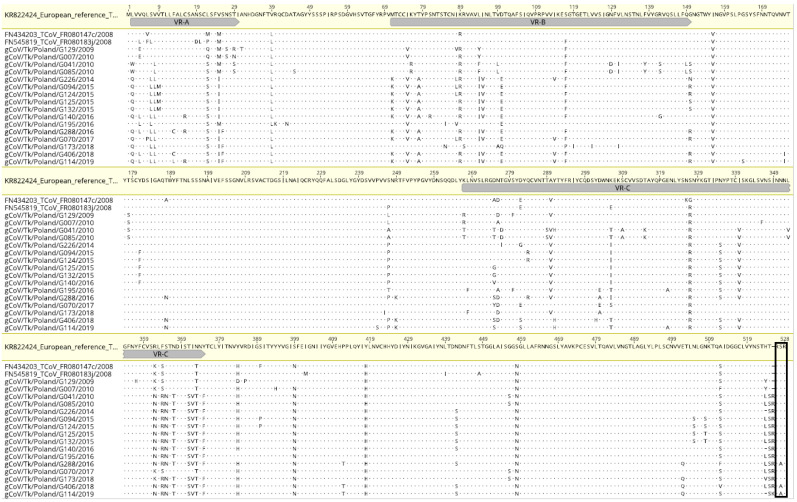
Amino acid alignment of the S1 subunit of Polish TCoVs and two other strains from France in comparison with European reference 080385d/France/2008 (KR822424) strain. Markings: dots—amino acids identical with the reference; gray bar beneath the reference strain—three variable regions (VR); box—putative cleavage site.

Similar tree topology was obtained in the case of the phylogeny of the amino acid sequences of the S protein. The consensus motif RXRR/X (X means serine in 12 TCoVs, and alanine in 3 strains, R-arginine, /-cleavage position) was found at the cleavage site of the S1 and S2 subunits in 15 TCoV Polish strains, except for G085/2010, in which the cleavage site was followed by the FTP amino acids sequence. Another cleavage site, resembling the furin-dependent one in IBV, was detected in the S2 subunit in all Polish and French TCoV strains (PQGR/S). Amino acids homology between Polish TCoVs was 93.8–100%. However, a comparison of the S1 protein resulted in an 89.8–100% homology (90.5–94.9% to the French reference TCoV) and S2 protein—96.3–100% (96.9–98.2% to the French reference TCoV). The alignment of the S1 protein of Polish and French TCoVs showed three regions with particularly high numbers of altered amino acid residues within them (Figure 2). Sequence comparison revealed low amino acid identity in these three regions: VR-A between amino acid positions 2 and 30 with a homology of 65.5–100%; VR-B between positions 71 and 150 with a homology of 79.0–100%; and a third VR-C between the positions 267–375 with a homology of 80.7–100%. The amino acid similarity of the fragments between these three regions was 93–100%.

### 3.3. Recombination Analysis

**Figure 3:**
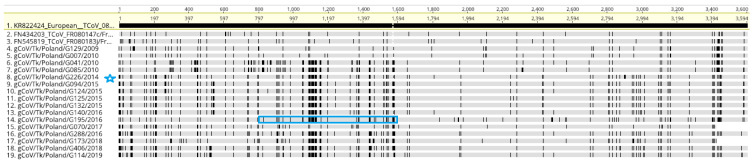
This is a figure alignment of the S gene sequences of Polish TCoVs and two other strains from France performed using MAFFT. The S gene sequence of TCoV 080385d/France/2008 (KR822424) was used as the reference sequence. Vertical lines indicate the single nucleotide polymorphism compared with the reference sequence. Blue box = recombination of G195/2016 with G226/2014 (strain marked with a blue star).

All European TCoV sequences were examined for recombination and the analysis of the data showed (Figure 3) that one sequence, G195/2016, recombined with a G226/2014-like virus (in the 768-nt fragment between nt 848 and nt 1616, blue box). This recombination event meets previously set conditions, as it was supported by four different methods (RDP, BootScan, SiScan and 3Seq).

### 3.4. Analysis of Selection Pressures

The selection pressure profiles of the S protein of Polish TCoV strains were analyzed. The calculated dN/dS ratio was 0.261, indicating that the S protein of these strains had evolved under negative selection. However, five individual codons were found under positive selection, which was located in the S1 protein. These residues were in the following positions: 4 (V/L/E), 275 (S/D/G/V/Y/T/A), 276 (D/N/A), 364 (R/S/F) and 512 (F/V/S/A) (*p*-value < 0.1), and were within the above distinguished VRs: VR-A (1 amino acid residue) and VR-C (3 residues).

## 4. Discussion

In this study, we performed molecular epidemiology of TCoV strains circulating in commercial turkey over a twelve-year period, from 2008 to 2019. The presence of turkey coronavirus in 10% (65/648) of the tested Polish flocks was found. Furthermore, such an occurrence of TCoVs seems to be lower when compared to the reported prevalence in turkey flocks in other countries. The monitoring of enteric turkeys in France showed the presence of coronaviruses in 37% of the tested intestinal samples [3]. Villareal et al. [6] demonstrated the presence of TCoV in 82.4% of the studied diseased turkey flocks, but also in one apparently normal. In subsequent studies of Brazilian poultry, the monitoring of diseased and healthy turkey flocks detected TCoVs in 71.1% and 28.6% of them, respectively [25]. A higher prevalence of TCoVs, more than 82% in enteric turkey flocks, was confirmed in other studies in Brazil [26]. In some studies conducted in the United States, the continuous circulation of TCoV was also reported [2]. On the other hand, no presence of coronaviruses was found in molecular monitoring in different regions of the United States [27,28,29]. However, the seroprevalence of this virus in commercial flocks was above 64% [30]. Furthermore, a similar seroprevalence of almost 74% in breeder turkeys and 60% in meat-type animals in Canadian commercial flocks were noted [31]. TCoV was also not detected in studies of healthy and diseased turkey flocks in Turkey [32]. The TCoV prevalence results obtained in the presented study have already been partially analyzed for the statistical relationship between the prevalence of four enteric viruses (astrovirus, coronavirus, parvovirus and rotavirus) in meat-type turkey flocks with the health status and the age of birds. At that time of the study, samples from both healthy and enteric turkey flocks were examined from 2008 to 2010; however, no correlation was found then, between the presence of turkey coronaviruses and the health status or age of the birds [33]. Such results are surprising, as the recent experimental results of Brown et al. (2019) have shown that even minute amounts of the virus are sufficient to initiate the disease in the flock [34]. On the other hand, to date, information on the S gene characteristics of European TCoV strains is scarce and concerns three isolates from 2008 from France. Thus, the presented investigation on the molecular feature of the S gene of TCoVs circulating in the turkey population in Poland contributes interesting data to the current state of knowledge.

As could be expected, S gene sequences of Polish TCoVs exhibited features of the French TcoVs, sharing with the European reference 080385d strain a 93.7–96.3% amino acid identity. This homology is even higher (96.9–98.2%) when the S2 subunit is compared, yet lower (90.5–94.9%) in the case of the S1 subunit. As presented in Figure 2, such divergence is especially visible in three regions of S1 protein, designated as VR-A (positions 2 to 30), VR-B (positions 71 to 150) and VR-C (positions 267 to 375), with only a 69.0–86.2%, 86.3–93.8% and 81.7–93.6% amino acid identity to the European reference TCoV, respectively. Furthermore, these regions more or less resembled highly variable regions of IBV, HVR1 (positions 38 to 67), HVR2 (positions 91 to 141) and HVR3 (positions 274 to 387), which were associated with neutralizing epitopes [35]. One of the proposed regions, namely VR-B, contains the region of sequence with the most variation identified in the North American TcoVs, according to Chen et al. [36]. A thorough analysis of the S gene sequences of 24 North American field isolates of TCoVs from 1994 to 2010, revealed one HVR in positions 126 to 134 at the amino-terminus S1a (1–204 in TCoV/IN/540/94) of their S1 protein. Moreover, two variable regions, identified in Polish TCoVs (VR-A and VR-B), are within the frame of the S1a region of the North American TCoVs. The comparison of amino acid sequences of these TCoV isolates showed that the sequence identity ranged from 77.6 to 96.6% for the S1a subunit that contains HVR, and 92.1 to 99.3% for the S2 subunit. In turn, such a highly variable S1a region in French turkey coronaviruses was proposed between positions 1 and 196 due to the identified low amino acid sequence identity (only 18%) of the French TCoVs compared to the North American ones as well as to quail coronavirus from Italy [11]. Region S1a also contains two variable regions proposed here, VR-A and VR-B. Our analysis shows the presence of an additional variable region, VR-C, located between positions 267 and 375. Moreover, although the selection pressure analysis showed that the TCoV strains were evolving under negative selection, some sites of the S1 subunit are positively selected, and of the five such sites, three are located in the third proposed VR-C region. Previous studies on the selection profile of North American IBV strains showed a number of positively selected amino acid sites, with some of them confirmed in vitro as characteristic of antigenic escape mutants. Interestingly, they were located in the HVR3 part of the IBV [37,38,39]. Regions with high amino acid variability may indicate the existence of antigenic differences between Polish viruses, similar to HVRs of IBV. Different serotypes of IBV may differ by 20% to 25% at the genomic scale, and up to 50% of amino acids in the S1 protein [40]. On the other hand, North American TCoV isolates (TCoV/VA-74/03, TX/1038/98, and IN/517/94), despite the high level of amino acid sequence identity (from 96 to 98%), belonged to different serotypes [16]. The answer to whether the Polish TCoV strains belong to different serotypes would ultimately clarify the cross-neutralization studies, which in turn would require their propagation in vitro and experiments in vivo to get neutralization sera.

A phylogenetic tree, constructed using the full-length S gene of the TCoV strains circulating in Poland over 12 years period, has revealed two main clusters: one formed by the early isolates identified at the beginning of the TCoV infection monitoring (from 2009 to 2010), and the other, formed by more recent strains from 2014 to 2019. Interestingly, together with the early Polish strains grouped the French ones from 2008 [11]. In the case of one Polish strain (G007/2010), the epidemiological interview indicated that the turkey poults came from the hatchery in France. It is interesting, however, whether the French-like TCoVs are the result of the introduction of the virus from France or if they were circulating over such a large area of western and central Europe. On the other hand, Chen et al. (2015) observed that TCoV isolates originating from the same US state clustered closely on the phylogenetic tree and concluded that distinct TCoV genotypes circulate endemically in various geographic locations. The S gene of two TCoVs from Minnesota identified 20 years apart, were 99.3% identical, implying that these isolates remained endemic and that no substantial genetic changes occurred over two decades [36]. It appears that such an endemic circulation of TCoV strains could be aided by the unique route of oro-fecal transmission and the lack of airborne transmission that could spread strains over greater distances [34]. Our analysis also showed that the S gene of Polish TCoV is not affected by positive selection, which confirms the conservative nature of these strains. It is also important to remember that TCoV remains infectious below the level of detection by molecular diagnostics, meaning that animals that tested negative prior to movement may be carrying an infectious virus [34]. Given the above, the hypothesis of the introduction of these strains as the result of the trade exchange seems more probable than the endemic circulation of such TCoV French-like strains.

The emergence of TCoV in North America and Europe was the result of recombination between genomes of circulating IBV strains and an unknown coronavirus, which was an S gene donor [9,16]. Moreover, the deep molecular analysis suggested that these donors on both continents were different coronaviruses [9]. A number of studies indicate the existence of a large abundance of coronaviruses, i.e., in the wild bird population, which seems to be a natural candidate for these donors [41,42]. Our sequence analysis also showed one TCoV strain had recombined with another one in the S1 gene. On the other hand, such recombination events between different IBV strains have already been shown many times in the field, so it cannot be ruled out that such events also happen in TCoV [43,44].

## 5. Conclusions

In summary, the circulation of turkey coronaviruses in Polish turkey flocks was identified. The monitoring of turkey flocks over a 10-year period showed a 10% prevalence of these infections in Poland. However, given the small time window for virus RNA detection (about 10 days) compared to antibody detection (6 weeks or more), it can be assumed by determining the presence of these viruses using specific serological tests (not currently available), that the results obtained would indicate a higher prevalence. The identified turkey coronaviruses were related in terms of the S gene structure to other European TCoVs. However, they were found to be genetically variable based on the sequence analysis reported here. The most recently identified TCoV strains belonged to a different cluster than the early Polish and French ones. Our analysis of the changes observed in the deduced amino acids of the S1 protein suggests the existence of several variable regions.

## Figures and Tables

**Table 1:** Prevalence of TCoV infections detected in samples from all 648 commercial turkey flocks collected between 2008–2019.

Year	No of Studied Flocks	No of TCoV-Positive	%
2008	80	5	6.3
2009	56	5	8.9
2010	32	10	31.3
2011	39	0	0.0
2012	44	3	6.8
2013	54	1	1.9
2014	77	5	6.5
2015	61	7	11.5
2016	53	7	13.2
2017	55	6	10.9
2018	52	7	13.5
2019	45	9	20.0
**Total**	**648**	**65**	**10.0**

**Table 2:** Sample information for TCoV sequences included in the phylogenetic analysis.

No	Isolate Name	Sample Collection	Age (Days)	Voivodeship of Origin	Genbank No
1	gCoV/Tk/Poland/G129/2009	November 2009	14	Wielkopolskie	ON227454
2	gCoV/Tk/Poland/G007/2010	January 2010	35	Wielkopolskie	ON227455
3	gCoV/Tk/Poland/G041/2010	April 2010	14	Warmińsko-Mazurskie	ON227456
4	gCoV/Tk/Poland/G085/2010	June 2010	91	Warmińsko-Mazurskie	ON227457
5	gCoV/Tk/Poland/G226/2014	November 2014	11	Warmińsko-Mazurskie	ON227458
6	gCoV/Tk/Poland/G132/2015	July 2015	24	Warmińsko-Mazurskie	ON227459
7	gCoV/Tk/Poland/G094/2015	April 2015	28	Warmińsko-Mazurskie	ON227460
8	gCoV/Tk/Poland/G124/2015	July 2015	49	Warmińsko-Mazurskie	ON227461
9	gCoV/Tk/Poland/G125/2015	July 2015	n/a	Warmińsko-Mazurskie	ON228954
10	gCoV/Tk/Poland/G195/2016	February 2016	150	n/a	ON227462
11	gCoV/Tk/Poland/G140/2016	May 2016	n/a	Warmińsko-Mazurskie	ON227463
12	gCoV/Tk/Poland/G288/2016	November 2016	20	Mazowieckie	ON227464
13	gCoV/Tk/Poland/G070/2017	March 2017	n/a	n/a	ON227465
14	gCoV/Tk/Poland/G173/2018	May 2018	14	Lubuskie	ON227466
15	gCoV/Tk/Poland/G406/2018	August 2018	21	Mazowieckie	ON246162
16	gCoV/Tk/Poland/G114/2019	March 2019	28	Mazowieckie	ON246163

**Table 3:** Sequence identity of S gene of Polish TCoVs and European reference strains from France.

	G288/2016	G406/2018	G114/2019	G094/2015	G124/2015	G125/2015	G132/2015	G140/2016	G226/2014	G173/2018	G070/2017	FR080147c/2008	FR080183j/2008	Eur ref	G007/2010	G129/2009	G041/2010	G085/2010	G195/2016
G288/2016	X	99.4	98.9	98.6	98.5	98.6	98.6	98.6	98.5	97.3	96.8	95.1	95.0	94.9	95.0	94.9	95.4	95.3	95.5
G406/2018	99.4	X	99.0	98.3	98.2	98.3	98.4	98.3	98.4	97.0	96.7	94.9	94.9	94.8	94.8	94.6	95.2	95.1	95.3
G114/2019	98.9	99.0	X	98.0	97.9	98.0	98.0	98.2	98.2	96.8	96.4	94.7	94.8	94.7	94.6	94.5	95.0	94.8	95.0
G094/2015	98.6	98.3	98.0	X	100.0	99.8	99.8	99.4	99.4	97.6	97.1	95.4	95.4	95.2	95.2	95.2	95.8	95.7	96.0
G124/2015	98.5	98.2	97.9	100.0	X	99.7	99.7	99.3	99.3	97.5	97.1	95.3	95.3	95.1	95.2	95.1	95.7	95.6	96.0
G125/2015	98.6	98.3	98.0	99.8	99.7	X	100.0	99.4	99.4	97.7	97.1	95.5	95.4	95.3	95.2	95.2	95.8	95.8	96.0
G132/2015	98.6	98.4	98.0	99.8	99.7	100.0	X	99.5	99.4	97.7	97.1	95.5	95.4	95.3	95.3	95.2	95.9	95.8	96.0
G140/2016	98.6	98.3	98.2	99.4	99.3	99.4	99.5	X	99.4	97.6	97.0	95.5	95.6	95.3	95.4	95.3	95.8	95.7	95.9
G226/2014	98.5	98.4	98.2	99.4	99.3	99.4	99.4	99.4	X	97.6	97.1	95.5	95.5	95.4	95.4	95.3	95.8	95.7	96.0
G173/2018	97.3	97.0	96.8	97.6	97.5	97.7	97.7	97.6	97.6	X	96.5	95.5	95.3	95.5	95.2	95.2	95.5	95.5	95.5
G070/2017	96.8	96.7	96.4	97.1	97.1	97.1	97.1	97.0	97.1	96.5	X	96.6	96.4	96.2	96.4	96.3	95.8	95.7	95.5
FR080147c/2008	95.1	94.9	94.7	95.4	95.3	95.5	95.5	95.5	95.5	95.5	96.6	X	98.2	97.8	97.6	97.5	95.9	95.8	95.3
FR080183j/2008	95.0	94.9	94.8	95.4	95.3	95.4	95.4	95.6	95.5	95.3	96.4	98.2	X	97.7	97.8	97.8	95.7	95.6	95.5
Eur ref	94.9	94.8	94.7	95.2	95.1	95.3	95.3	95.3	95.4	95.5	96.2	97.8	97.7	X	97.2	97.1	95.4	95.3	95.2
G007/2010	95.0	94.8	94.6	95.2	95.2	95.2	95.3	95.4	95.4	95.2	96.4	97.6	97.8	97.2	X	99.5	95.9	95.8	95.2
G129/2009	94.9	94.6	94.5	95.2	95.1	95.2	95.2	95.3	95.3	95.2	96.3	97.5	97.8	97.1	99.5	X	95.8	95.7	95.3
G041/2010	95.4	95.2	95.0	95.8	95.7	95.8	95.9	95.8	95.8	95.5	95.8	95.9	95.7	95.4	95.9	95.8	X	99.7	95.3
G085/2010	95.3	95.1	94.8	95.7	95.6	95.8	95.8	95.7	95.7	95.5	95.7	95.8	95.6	95.3	95.8	95.7	99.7	X	95.1
G195/2016	95.5	95.3	95.0	96.0	96.0	96.0	96.0	95.9	96.0	95.5	95.5	95.3	95.5	95.2	95.2	95.3	95.3	95.1	X

Shaded fields indicate nucleotide identity between Polish and those available in the GenBank French TCoV strains from a given phylogenetic Group 1 and 2. (dark grey—between strains of Group 1, light grey—between strains of Group 2)

## Data Availability

The genome sequences generated in this study were submitted to the GenBank database (https://www.ncbi.nlm.nih.gov/genbank/ accessed on 14 April 2022) under accession numbers ON227454-ON227477 and ON ON228954-ON ON228955.
